# Feature Extraction for Finger-Vein-Based Identity Recognition

**DOI:** 10.3390/jimaging7050089

**Published:** 2021-05-15

**Authors:** George K. Sidiropoulos, Polixeni Kiratsa, Petros Chatzipetrou, George A. Papakostas

**Affiliations:** HUMAIN-Lab, Department of Computer Science, International Hellenic University, 654 04 Kavala, Greece; georsidi@teiemt.gr (G.K.S.); polykyra1@teiemt.gr (P.K.); peyhatz@teiemt.gr (P.C.)

**Keywords:** biometrics, finger vein recognition, identity recognition, feature extraction, deep learning

## Abstract

This paper aims to provide a brief review of the feature extraction methods applied for finger vein recognition. The presented study is designed in a systematic way in order to bring light to the scientific interest for biometric systems based on finger vein biometric features. The analysis spans over a period of 13 years (from 2008 to 2020). The examined feature extraction algorithms are clustered into five categories and are presented in a qualitative manner by focusing mainly on the techniques applied to represent the features of the finger veins that uniquely prove a human’s identity. In addition, the case of non-handcrafted features learned in a deep learning framework is also examined. The conducted literature analysis revealed the increased interest in finger vein biometric systems as well as the high diversity of different feature extraction methods proposed over the past several years. However, last year this interest shifted to the application of Convolutional Neural Networks following the general trend of applying deep learning models in a range of disciplines. Finally, yet importantly, this work highlights the limitations of the existing feature extraction methods and describes the research actions needed to face the identified challenges.

## 1. Introduction

Identity verification has become an integral part of people’s daily life. Logging into computers or electronic accounts, using ATMs (Automated Teller Machines), and being given entrance permission to a bank or an area generally are just some of the most common cases where identity verification is needed. There are many ways to verify someone’s identity. The usage of a password is the most popular, but it tends to be obsolete, as biometrics seem to be the key to the person identification problem.

Biometrics refers to metrics related to human characteristics. Biometric identifiers are the distinctive, measurable characteristics used to label and describe individuals. They are usually divided into two categories: (1) *behavioral*, such as typing rhythm, gait, and voice; and (2) *physiological*, e.g., fingerprints, face, iris, and finger vein. Each category has both advantages and disadvantages and some of them are already being used extensively.

*Finger vein recognition* is a relatively new method of biometric authentication. It matches the vascular pattern in an individual’s finger to previously obtained data. Finger vein biometrics is a field that is currently in the spotlight. Because it is rather new compared with other biometric fields, the information in addition to the conducted studies is poor. The advantages of using the veins of the fingers as biometrics are fair enough and constitute the main motivation for applying this technology. Firstly, it is a biometric trait that is difficult to forge, as the main functionality is to emit infrared light in the finger and capture via a camera the shape of the finger’s veins. It is well known that the shape of the finger vein is unique in humans, which makes a finger vein a very good means of identification. Other advantages of the unique identification the finger vein offers are that it is achievable through any finger of a human and the vein patterns are permanent, meaning that they remain unchanged over time and can be measured without subjecting the human to a painful process. The finger vein trait satisfies in some degree the seven factors [1] that define the suitability of a biometric trait in order to be useful for identity authentication: (1) Universality, (2) Uniqueness, (3) Permanence, (4) Measurability, (5) Performance, (6) Acceptability, and (7) Circumvention. As a result, finger vein biometrics has gained ground due to all these advantages and raises the interest for most researchers to conduct studies in this field.

The standard method used to acquire finger vein images includes the position of a CCD or CMOS camera opposite to a near-infrared (NIR) light source (LED), with the finger inserted between them. Due to the property of the hemoglobin having a lower absorbance to the NIR wavelengths than the visible ones, the camera can capture an image containing the finger veins. Of course, the wavelength of the used LED source affects the representation quality of the vein patterns. The captured vein patterns are compared with prototype veins stored in a smart card. The images that are taken include not only vein patterns but also irregular shading and noise due to the varying thickness of finger bones and muscles. Therefore, the most challenging part of the whole process is to use the right method to extract the finger vein features.

**Figure 1:**
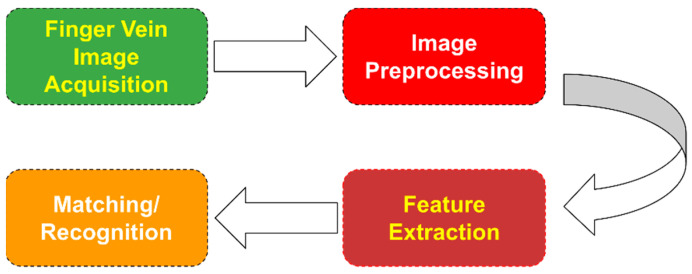
Finger vein feature extraction flowchart.

Figure 1 shows a flowchart that describes the steps a typical finger vein recognition algorithm follows. The finger images are usually taken by using a camera and a separate illumination source emitting near-infrared light. *Image preprocessing* usually includes denoising (such as image smoothing, blurring, etc.), image thresholding, image enhancement, and skeletonization. The next step, known as *feature extraction*, is the one that is going to be studied in the following sections thoroughly. Finally, in the last step, the extracted features are used as inputs in a *matching/recognition* model.

A plethora of methods have been proposed so far for feature extraction, including the usage of, e.g., templates, transformations, minutiae, line tracking, binary feature extraction, histogram analysis, and mathematical procedures. This work aims to contribute in three distinct directions: (1) it systematically reviews the feature extraction methods proposed in the literature in the last several years; (2) it identifies the shortcomings of the state-of-the-art methods; and (3) it sheds light on the current challenges that need to be addressed by the scientific community towards the development of more efficient finger vein recognition systems.

The structure of this paper is as follows. Section 2 highlights the novelties of this study in comparison with similar works published in the past. Section 3 analyzes the literature in order to investigate the scientific interest in finger vein recognition over the last 13 years. Section 4 presents a categorization of the feature extraction methods that helps us study the main characteristics of the reviewed methodologies. Section 5 points out the evolution from feature extraction to feature learning in the framework of deep learning. Section 6 summarizes the main identified implementation aspects. Finally, Section 7 discusses the limitations of the reviewed feature extraction methods, identifies the challenges that need to be addressed, and concludes the entire study.

## 2. Related Work

In the literature, few works that review finger vein biometrics have been presented. The first review paper was published in 2014 by Yang et al. [2], focusing on the techniques employed in image acquisition, the public finger vein databases, and the applied feature extraction methods. From the feature extraction point of view, the presented study was based on an analysis of only 15 papers, a very small fraction of the works published until 2014. Therefore, the importance of this first review paper was not the review of all the related published papers, but the initiation of a systematic scientific discussion regarding finger vein recognition.

Two years later, Syazana-Itqan et al. [3] presented a review on finger vein biometric approaches. This review paper discussed published methods in preprocessing, feature extraction, and classification and surveyed some of the existing finger vein databases. The presented study derived from an analysis of 18 papers proposing conventional methodologies, 5 papers that used Machine Learning (ML) approaches, and 1 paper that introduced Convolutional Neural Networks into finger vein recognition. This second review paper also did not present all of the publications until 2016 but focused on a small part of the literature.

A year after the last review publication, Dev and Khanam [4] published a conference paper that reviewed the feature extraction methods applied in finger vein recognition. The data of this study included 26 papers published in the period 2004–2015. Although the number of reviewed publications was higher than that in the previous review papers, this work for the first time categorized the feature extraction methods into three categories in order to present the methods in a more systematic way.

Another recent review paper for finger vein recognition was published in 2019 by Shaheed et al. [5]. This work is the most complete study in finger vein biometrics since it reviews the available datasets, the feature extraction methods and their performance, and spoofing attacks. Regarding the analysis of the feature extraction methods, this paper reviewed 25 publications presenting conventional methodologies and 8 and 9 publications that used machine learning and deep learning models, respectively.

The next year, two review papers were published on finger vein recognition, with the first one by Mohsin et al. [6], who conducted a review with articles from three different databases and divided them into two categories, namely software and hardware-based systems. The specific review presented the trends and focus of the literature regarding the technical and hardware problems, proposing solutions for the security problems of the systems, presenting some available databases, and discussing the motivations, challenges, recommendations, and future directions of the field. On the other hand, F. Elahee et al. [7] compared the results of studies that used deep learning for the authentication process by presenting their results in a comparative manner.

This work aims to complement the previous four review papers along three directions: (1) it extends the literature analysis to a wider (13-year) period (between 2008 and 2020); (2) it focuses only on the feature extraction methodologies; (3) it reviews many more publications (96 papers) proposing conventional feature extraction algorithms; and (4) it reviews 14 deep learning methodologies that replaced feature extraction with feature learning. The examined feature extraction methods are clustered into some categories based on the common feature extraction principles they share. Moreover, their performance is not studied in this work, since each method has different performance indices and the experiments were conducted with different datasets and different classifiers; thus, no useful conclusions can be derived.

## 3. Literature Analysis

As is mentioned in the introduction section, the main goal of this section is the justification of the continuously increasing scientific interest in finger vein feature extraction methods.

**Figure 2:**
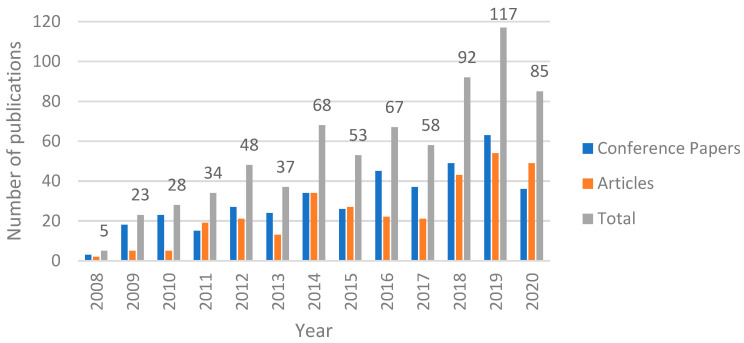
Number of finger-vein-related publications of Scopus per year for the last 13 years.

**Figure 3:**
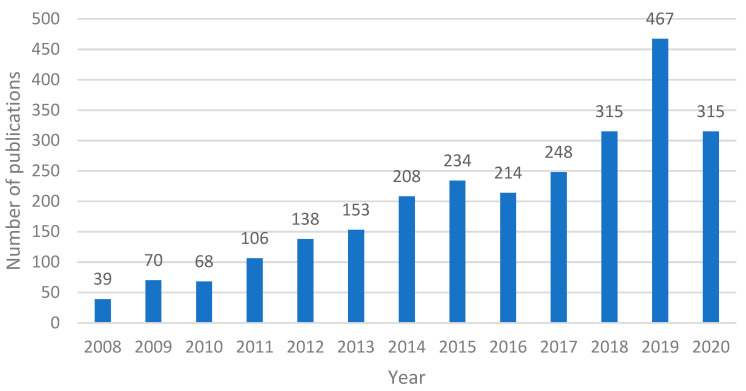
Number of finger-vein-related publications of Google Scholar per year for the last 13 years.

The analysis of the literature for finding and counting the number of publications during a certain period constitutes a laborious task. However, for this study, we decided to make use of two different well-known publication abstract and citation databases. The first is the Scopus bibliographic database [8], which is commonly accepted by the scientific community and includes quite enough information for our analysis. The second is the Google Scholar [9] search engine, which is broader than Scopus as it contains publications from a wide range of publishing houses compared with Scopus, which includes only publications evaluated for inclusion in this dataset. Thus, in order to be more specific to our analysis, we refer to that as well. The search was performed by applying a rule with the keywords **“finger vein” AND (recognition OR authentication)** for the Scopus database and **“finger vein recognition” OR “finger vein authentication”** for the Google Scholar network. It is worth mentioning that the Scopus database provides information (Figure 2) regarding the type of publication (journal or conference paper), while this information is not easily extracted from Google Scholar (Figure 3) and in this case the publications are only presented cumulatively.

The period of our analysis was set to 13 years, from 2008 to 2020, since these years include the most publications in this research field and research in the current year is ongoing. Moreover, we were only interested in two types of publications, namely Articles (journal publications) and Conference papers, while our subject area of interest is Computer Science and Engineering in both cases.

Figure 2 and Figure 3 illustrate the number of papers published in the last 13 years, with a time step equal to 1 year. From Figure 2, the upward trend of the interest in finger vein feature extraction is obvious, with the 5-year period 2008–2012 being characterized by a rapid increase in all types of publications. However, publication seems to fall a bit in 2013, followed by an increase in 2014. This rise and fall can be seen for six consecutive years, starting from 2012 and ending in 2017. Years 2018 and 2019 saw a large increase, with the highest number of publications compared with all previous years, with the last year having a decreased number of publications, although it is still higher than the average number of publications during the last few years. Moreover, the year 2020 was a peculiar year because of the COVID-19 pandemic, during which the research efforts in all disciplines were reduced worldwide.

By focusing our analysis on the time period of the last 13 years we also derived that 2019 was the most productive year in the history of research on finger vein feature extraction methods, concerning the Scopus library, during which 63 conference papers and 54 articles were published. This number is quite large considering the high competitiveness occurring in other fields of person identification and reflects the amplification of the engagement of new scientists with finger vein feature extraction-related topics. Another remarkable characteristic is number of conference papers showing an upward trend compared with articles. The same can be derived concerning the Google Scholar database (Figure 3), as the year 2019 is the most productive year as well, with 467 papers in total. The difference in this case is that, with the exception of 2010 and 2016, where there was a slight drop in publications, there has been a steady increase in the interest in finger vein publications throughout the years. The last year, 2020, saw a quite high drop in terms of publications in both cases, where the number dropped by about 30% in both cases.

Conclusively, it can be stated that the research in the field of finger vein feature extraction has experienced its highest evolution so far. The outcomes of this study should be translated to more research activities since time and the large amount of prior knowledge favor the discovery and development of next-generation frameworks in both finger vein feature extraction theory and applications.

## 4. Finger Vein Feature Extraction

For the needs of the study, the analyzed feature extraction algorithms were divided into five categories: (1) algorithms based on vein patterns, (2) algorithms based on dimensionality reduction, (3) algorithms based on local binary patterns, (4) algorithms based on image transformations, and (5) other feature extraction methods. For each category, a cumulative and comparative table is presented for the methodologies belonging to the specific category. It should be noted that, in most cases, the feature extraction method’s performance is evaluated according to the Recognition Rate (RR), Accuracy, and Equal Error Rate (EER) metrics. In our case, we regarded the performance of a methodology as having a high RR or accuracy when its performance was equal to or higher than 99%, and its performance in terms of the EER was regarded as low when it was lower than 1%. The selection of these thresholds was based on the high demands imposed by the critical application of the biometric systems.

A finger vein image is acquired by placing a finger on a camera, with a near-infrared (NIR) light pointed towards it from the opposite side of the camera. With the NIR light pointing towards the finger, the veins become visible and thus a feature extraction process can be applied.

### 4.1. Feature Extraction Based on Vein Patterns

**Figure 4:**
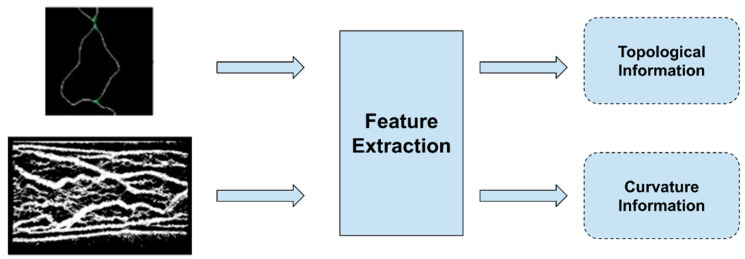
Typical extraction of information relative to the vein patterns.

A typical process followed for the extraction of vein patterns is shown in Figure 4. In most cases, the resulting image, after the preprocessing step and just before the feature extraction step, is shown in the figure. Then, the features that are extracted from these kinds of images, in most cases, focus on the topological or curvature information of the veins.

Starting in 2004, Miura et al. [10] used the repeated line tracking algorithm to extract features from finger vein images. Miura et al. [11], three years later, proposed another method that utilized the fact that a vein appears like a dent in the cross-sectional profile; thus, it is checked and the centerlines of veins are emphasized.

In 2009, Choi et al. [12] used a principal curvature, which is obtained from the eigenvalues at each pixel of the Hessian matrix for the finger vein feature extraction. Later, Yu et al. [13], in their method, used normalization of the finger vein image, orientation estimation, and Gabor filtering, then image segmentation and image thinning, and, finally, minutiae point extraction of the image. From this combination, bifurcation points and ending points are extracted from vein patterns and used as geometric representations of the shape of the vein patterns. Finally, the Hausdorff distance algorithm is used to identify possible positions of the vein pattern shape. Because the original Hausdorff distance (HD) algorithm is sensitive to small perturbations, the modified Hausdorff distance (MHD) algorithm is deployed. In the same year, Yang proposed two different feature extraction methods. Starting with [14], Yang et al. proposed a method that used the circular Gabor filter as well as multi-channel Gabor filters to produce vein vessel information. They extracted and combined the local moment, topological structure, and vein-shape features from the finger vein images. In [15], Yang et al. used a bank of seven symmetric Gabor filters to exploit vein information. Qian et al. [16] used the maximum curvature to extract the finger veins from the images. The extracted finger veins are then skeletonized and a deblurring process is performed on the skeletonized image, which is used as the feature vector of the finger vein. A year later, Kejun et al. [17] also used 2-D Gabor filters to extract phase and direction features.

In 2011, Song et al. [18] proposed the mean curvature method, which finds valley-like structures with negative mean curvatures. In the same year, Yang et al. [19] proposed another method that used Gabor filters because they are tunable in scale and orientation. Considering the variety of vessels in orientation and diameter, Gabor filters are suitable for region texture analysis. For local finger vein codes (L-FVCodes) with the scale being equal to 2 (s = 2), the FRR for the forefinger was 1.6%, while for a scale equal to 3 (s = 3) the FRR for the forefinger was 2. A year later, following the same approach of using Gabor filters, a number of methods were proposed. Xie et al. [20] described a guided Gabor filter, which is an appropriate method for poor-quality images.

In 2013, Venckauskas and Nanevicius [21] used the pattern of the finger veins to generate a cryptographic key that corresponds to the specific finger vein. Firstly, the starting, ending, and vein crossing points are determined. After determining these points, a contour tracing algorithm is applied, with the starting point for contour tracing being selected by the user. Using the contour tracing algorithm, the traced contours are used to generate a partial cryptographic key and all of the partial keys generated are combined into one key. Prabhakar and Thomas [22] applied the maximum curvature feature extraction method to provide robustness on the vein width and brightness variations and a postprocessing method to eliminate false minutiae points.

Nivas et al. [23] used the repeated line tracking algorithm. In the same year, Mohammed et al. [24] also used the repeated line tracking algorithm for their multi-model identification, which combined iris and finger vein recognition. On the other hand, Liu et al. [25] proposed a minutiae matching method based on singular value decomposition (SVD), which consists of three distinct processing steps: minutia pairing, false pair removal, and score calculating. For minutiae extraction, they used the bifurcation point (BP) and the ending point (EP) from skeletonized finger vein images. They extracted four different minutia features, including the coordinate value and three local descriptors, for minutiae matching. The local descriptors consist of the local average intensity (LAI), local intensity deviation (LID), and local extensive binary pattern (LEBP).

In 2015, Mantrao et al. [26] proposed a method that uses minutiae matching. In this method, after preprocessing the finger vein image, a skeletonized image of it is created and minutiae points are then extracted using morphological operations. Prasath et al. [27] proposed a method that combines the features extracted from sclera images and vein images from the fingers. The features extracted from the sclera images are extracted using the Y-shape feature extraction method and the features extracted from the vein images are extracted using the repeated line tracking method. Gupta et al. [28], in their method, use multi-scale match filtering to enhance the veins in the images obtained and line tracking to extract the veins. What this system does is iteratively determine the threshold surface and does not require any parameters, such as neighborhood size; thus, it can extract variable-width veins.

Bansal et al. [29] used minutiae extraction and curve analysis. For the minutiae points, they applied a thinning technique to extract the finger vein skeleton. Then, the minutiae points were computed and, in the last step, the minutiae coordinates were produced. For the curve analysis, they applied calculus methods to obtain the curves. Then, they found and counted the lines or the curves that connected two points and, in the last step, found the amplitude, phase, and actual curve length. Liu et al. [30] proposed a modified repeated line tracking algorithm, which figures out the locus space of a finger vein based on revised parameters.

In 2016, Kalaimathi et al. [31] proposed a feature extraction method in which a gradient-boosted feature algorithm is applied. Image gradients extract information from input datasets and then a gradient image is calculated from the default image with the use of the Sobel filter. Three parameters are taken into account in order for the algorithm to make a decision: scalability, integrity, and flexibility. After that, classification is performed with the use of a Support Vector Machine (SVM) model. Matsuda et al. [32] solved the problem of irregular shading and vein deformation in the captured finger vein image by using the curvature of image-intensity profiles for feature point extraction. Then, a vein-pattern map was calculated from the descriptor points using eigenvalues. Zou et al. [33] proposed a method that uses multiple samples of the same finger vein to create features. Each sample is segmented and all of them are overlaid. By removing the dots that appear only in one of the samples, a feature template is created. The number of samples that are taken affects the quality of the feature template.

In 2017, Brindha [34] also used minutiae extraction; however, in addition, a method for feature reduction by neighborhood elimination using the Euclidean distance was applied.

Babu et al. [35], in their work, used a Gabor filter to extract the texture of the finger vein since Gabor filters can be tuned to capture a finger vein image’s local orientation and frequency information. They applied Gabor filters with specified orientations and convolved them with the enhanced image to filter the unwanted regions. Next, a postprocessing task involving a morphological top-hat operation was applied to the extracted veins to further improve the quality of the vein patterns.

In 2018, Prommegger et al. [36] established a new finger vein dataset that includes videos of vein structures all around the finger. Additionally, they compared the performance of different feature extraction algorithms, namely maximum curvature, principal curvature, and Gabor filters. On the other hand, Yang et al. [37] proposed a feature extraction methodology that extracts the anatomical structure (directionality, continuity, width variability, smoothness, and solidness) of finger veins.

In 2019, Yang [38] proposed a finger vein code indexing and matching method. The indexing process includes the extraction of the vein patterns, the detection of the direction of each vein segment, and the construction of an elliptical direction map. Applying finger vein recognition in smart home security, Sarala et al. [39], after preprocessing the image, generated a binary image of the veins and created a feature vector that included the vein width, length, position, and intersection points. Ali et al. [40] developed the Straight Line Approximator (SLA) for feature space extension. After detecting the region of interest (ROI) and the finger vein with the maximum curvature method, they used the proposed SLA to extract features. For each sub-block of the image, the SLA method fits a line for the points inside the block, combines its slop and offset components, and eventually aggregates the components of all those blocks to create the feature vector. For a different application, Ilankumaran and Deisy [41] proposed a $C^{2}$ code, which was formulated by using the orientation and magnitude information extracted from finger vein and iris images. For the feature extraction, after extracting the ROI, enhancing the image, and applying two Gabor filters, the $C^{2}$ code scheme is applied for feature extraction. Yang et al. [42] proposed a new feature extraction method called Polarized depth-Weighted Binary Direction Coding (PWBDC) for feature extraction from dorsal finger vein and texture images. This method includes polarized direction extraction, extended normalized angular binary coding, and self-adaptive depth-dependent weighting.

In 2020, Yong [43] applied a curvature algorithm for feature extraction in their FPGA system by calculating the eigenvalues of the image’s Hessian matrix. Villar et al. [44] proposed the usage of Spectral Clustering (SC), in combination with a normalized Laplacian matrix and eigenvalues, to extract the vein patterns. The SC is applied on all the ROIs that are detected in the image through a mask application and the features are then used on a Logistic Regressor for classification.

In general, the methodologies that extract features regarding the patterns of the finger veins depend on the preprocessing steps to a high degree, as the more visible the veins are in the binary image, the better the performance of each methodology. The algorithms, in general, are efficient enough to be ported into an ARM or low-power device and have low EER values (lower than 1% in most cases). On the other hand, these algorithms require calibration by setting parameters that can influence their performance, and the classification is done via a matching score or distance/similarity calculation, which can be efficient for small databases, but time consuming in large ones. Moreover, Gabor filters seem to be a useful feature extractor of vein patterns due to their ability to describe the frequency and orientation of texture patterns. Table 1 sums up the studies mentioned in this category, showing the key features, advantages, and disadvantages of each method.

### 4.2. Feature Extraction Based on Dimensionality Reduction

In 2004, Beng and Rosdi [45] used a pattern map template to extract the features from finger vein images. The pattern map is generated by first choosing a random finger vein class and generating a mean image. Then, the mean image is sliced onto M blocks with the same width and height and then PCA is performed over all M blocks, resulting in *eigenveins*. The eigenvein with the maximum eigenvalue is chosen as a Gaussian low-pass filter and all the others as derivative filters.

In 2010, Liu et al. [46] used Orthogonal Neighborhood Preserving Projections for feature extraction and dimensionality reduction. It is a linear dimensionality technique that preserves both the local and global geometry of high-dimensional data samples. Guan et al. [47] proposed a different weighted bi-directional B2DPCA (WB2DPCA), called Bi-directional Weighted Modular B2DPCA (BWMB2DPCA), to overcome the problems of the finger position, uneven lighting from the infrared light, etc. According to the proposed method, the image is divided into sub-blocks and each sub-block is dealt with as a group of sub-image blocks. The rest of the steps are the same as in WB2DPCA.

The next year, Ushapriya [48] proposed a combination of PCA and a Radon transform for feature extraction. In that case, the features are derived by using the Radon projections of a finger vein image in different orientations and PCA is applied to each projection matrix. Wu et al. [49] used Principal Component Analysis (PCA) and Linear Discriminant Analysis (LDA). Firstly, PCA is applied for dimension reduction and then LDA is applied for feature extraction. The combination of these two methods gives better classification performance by reducing the amount of irrelevant and redundant information in the data.

In 2012, Yang et al. [50] used 2DPCA for extracting the features of an image. Damavandinejadmonfared et al. [51] used PCA, KPCA, and KECA to test the performance of a neural network with various numbers of training and testing images for each subject using each method. Two years later, Hajian et al. [52] used three different KPCAs (Polynomial, Gaussian, and Laplacian) to extract the features of the data.

In 2015, You et al. [53] proposed a combination of the 2DPCA and KMMC methods. In this work, 2DPCA was applied to the image in the horizontal direction, which was used as a training set. Van et al. [54] proposed a method in which Modified Finite Radon Transform (MFRAT) is applied to each pixel in the region of interest. Then, a grid sampling strategy is performed, which results in *m* sets of *n* pixels. Finally, GridPCA is performed on these sets to calculate the features of the finger vein.

The next year, Qiu et al. [55] used a dual sliding window to first find the phalangeal joint of the finger. Then, after enhancing the finger vein image, it is transformed using the proposed pseudo-elliptical sampling model. Lastly, a 2DPCA single direction algorithm is used on the transformed image to obtain the feature matrix.

In 2017, Xi et al. [56], after preprocessing the finger vein images, obtained class centers using the (2D)^2^PCA dimensionality reduction. Using the class centers, a relation matrix was calculated and transformed into binary code templates that were used as the features of the finger vein. In 2018, in their multi-biometric system, Yang et al. [57] applied 40 Gabor filters to an image, generating a real-valued vector that was then passed through to a Linear Discriminant Analysis dimensionality reduction technique.

In 2020, Hu et al. [58] applied a Multi-scale Uniform Local Binary Pattern block to extract local texture features, followed by the application of a (2D)^2^PCA method based on a block to preserve the local information of the finger vein images.

Dimensionality reduction methodologies, in most cases, use a variation of the PCA algorithm, which can reduce the feature vector to any desired length. As a result, this type of feature extraction can yield good results, and most studies had high accuracy rates. In general, these methodologies are combined with a type of matching function for the classification, yielding satisfactory results, or the authors trained a type of Machine Learning (ML) model to obtain higher accuracy rates. Moreover, dimensionality reduction algorithms have been applied in combination with other types of feature extraction methods and, as a result, depend heavily on them. Table 2 sums up the methodologies mentioned in this category, showing the key features of each one and their advantages and disadvantages.

### 4.3. Feature Extraction Based on Local Binary Patterns

**Figure 5:**
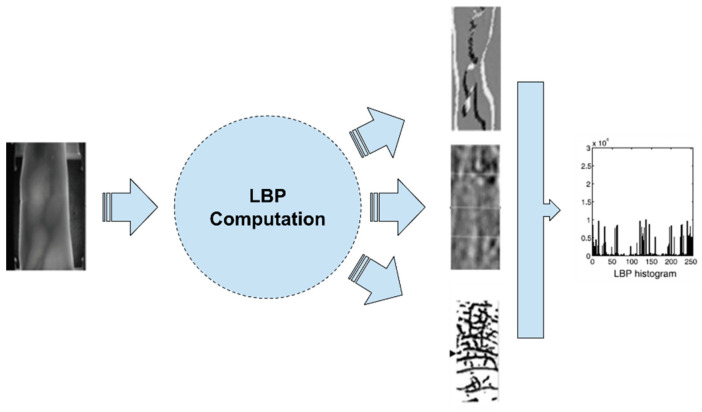
Feature extraction using LBP-based features.

In Figure 5, the extraction process of Local Binary Pattern (LBP)-based features is depicted. These methodologies, after extracting the ROI of the finger vein, apply a type of LBP-based algorithm and extract the LBP images as shown in the figure. In some cases, the histogram of the image is used for the matching process instead.

In 2006, Zhang et al. [59] proposed a multi-scale method based on curvelet and local interconnection structure neural networks. The former is used for enhancement while the latter is used to extract the features. It is stated that the proposed method solves the problem of how to extract features from obscure images.

Three years later, Lee et al. [60], after preprocessing the finger vein image, aligned the finger vein using an affine transformation and then extracted the LBP code.

In 2010, Lee et al. [61] proposed a method using a weighted LBP. Firstly, the LBP method was applied to extract the holistic codes without detecting the vein patterns by reducing the processing time caused by the vein detection. Secondly, the extracted LBP codes were used in combination with a SVM classifier to classify the local areas of vein patterns into three categories: (1) Large Amount (LA), (2) Medium Amount (MA), and (3) Small Amount (SA). Finally, based on the determined local area types (LA, MA, and SA), different weights were assigned to the extracted LBP codes of each local area type.

In 2011, Park [62] combined the LBP with the Gabor wavelet to extract local and global features from a finger vein image. Lee et al. [63] compared the LBP with the Local Derivative Pattern (LDP) in an attempt to overcome a problem that is related to local shadows appearing on the finger area when binarization is used. In 2012, Yang et al. [64] extracted the LBP code of finger vein images from two or more fingers of each user. Yang et al. [65] used the Personalized Best Bit Map (PBBM) for feature extraction. In 2014, Lu et al. [66] proposed a new local binary pattern (LBP) extraction method called Generalized Local Line Binary Pattern (GLLBP) to extract the features from finger veins.

In 2015, Dong et al. [67] transformed the weighted SLGS (W-SLGS) to MOW-SLGS. This algorithm makes clockwise and counterclockwise comparisons between the pixel values for a number of angles as the SLGS does. From the feature vectors that result, the maximum value is chosen as the feature of the target pixel and the weight of the pixel is set according to the distance between the pixels. The same year, William et al. [68] adopted LHBGC as a finger vein feature extractor. LHBGC differs from the BGC in enclosing not only texture but also magnitude information. The texture information encodes the local differences, while additional discriminant information is encoded by the magnitude components. The extracted information is divided equally into a set of cells. For each cell, the histogram is computed, with the magnitude components being the weight representation of the distribution of the texture values. Next, the histogram of each cell is vectorized and all the cells’ histogram vectors are concatenated. The extracted feature sets from each finger image are fused together into a super-vector based on a serial feature fusion technique. Khusnuliawati et al. [69] compared Scale-Invariant Feature Transform (SIFT) with the LEBP (along with LmBP and LdBP) for feature extraction using the LVQ classifier for the matching process. Dong et al. [70] extracted features using the Difference Symmetric Local Graph Structure (DSLGS) algorithm. This method considers a center pixel, which is the target pixel and 14 more pixels that surround the target one. Then, the difference value between the pixels is calculated to give the difference coefficient, which leads to stable feature extraction. To calculate the DSLGS, there are three steps.

In 2016, Yang et al. [71] used the Cross-Sectional Binary Code (CSBC) to extract the features from the finger vein and the finger dorsal texture and fuse them as one feature. In 2019, Liu et al. [72] utilized Pixel Difference Vectors (PDVs) for feature extraction and then used the Anchor-based Manifold Binary Pattern (AMBP) for the feature learning process. Extending their previous work, Liu et al. [73] developed a new local binary learning feature, called Personalized Binary Code (PBC), for which multiple directional PDVs are calculated for all the images of the training set of a class. Then, all the vectors are combined into one and the binary code is calculated. Then, a function is applied to the binary vector calculated to make it more compact, discriminative, and personalized. Lastly, Su et al. [74] used both finger vein and electrocardiogram (ECG) signals in their identification system, extracting LBP features from the former and combining them with other features extracted from the latter. Lastly, they projected the matrix with the improved binary vector into the low-dimension binary features, applied clustering of those features in a codebook using k-means, and, finally, created a histogram as the image representation. On the other hand, Lv et al. [75] combined the features extracted from their proposed Adaptive Radius LBP from images of both a fingerprint and a finger vein.

Local binary pattern-based methodologies have been proven to perform very well in finger vein authentication in general, with EERs lower than 0.1% in most studies. Their main advantage is that they are resistant to irregular shading and saturation from the image-capturing device. Additionally, depending on the implementation, they can be very fast and implemented in low-power devices. Table 3 summarizes the main characteristics of the methods in this category.

### 4.4. Feature Extraction Based on Image Transformations

**Figure 6:**
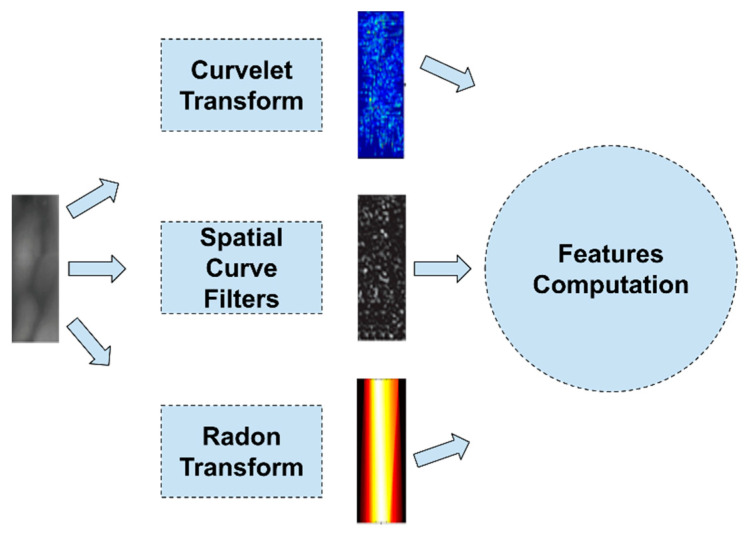
Feature extraction using image-transformation-based features.

In Figure 6, the extraction process of image-transformation-based features is depicted. These methodologies, after extracting the ROI of the finger vein, apply image transformation filters and for the prediction use a classifier-based prediction method, e.g., an ML model, or a matching rule.

In 2005, Zhang et al. [76] applied a multi-scale self-adaptive enhancement transformation to a finger vein image. Based on this method, the image is emphasized and noises are reduced. Consequently, different receptive fields are used to deal with different sizes of finger vein patterns. Moreover, the use of the integral image method makes this method very fast.

Four years later, Wu et al. [77] used the continuous Radon transform [78], leveraging its properties [79], for geometry transformation to extract finger vein features for the needs of a driver identification system, using a neural network for decision-making.

In 2014, Ramya et al. [80] used the Haar classifier and line detection to extract the features from a finger vein image. Sreekala et al. [81] used a second-generation wavelet transformation, after preprocessing the finger vein image, for feature extraction in their simulation of a security system. Gholami et al. [82] proposed a method that extracts veins with the use of entropy thresholding, applying the Radon transformation to images and resizing and partitioning them.

A year later, Santosh et al. [83] used the Discrete Wavelet Packet Transform (DWPT) to decompose finger vein images without computing the High-High (HH) sub-band as it contains the majority of the noise. The feature vector consisted of the averages and standard deviation of the energy of the image for each level of decomposition. Kejun et al. [84] introduced two discrete algorithms based on the Unequally Spaced Fast Fourier Transform (USFFT) and wrapping for the finger vein feature extraction. On the other hand, Shareef et al. [85] used the Haar wavelet moments as the features of the finger vein. In the first step, the image is divided into overlapping blocks and then a 2D wavelet transform is applied to the blocks three times. Then, the energy is computed for each wavelet sub-band of a block, which is used as a feature of a finger vein.

In 2016, Qin et al. [86] proposed a new approach to extract finger veins by detecting the valley-like structures based on the curvatures in Radon space. For each of the image’s pixels, eight patches centered on it were obtained after the rotation of a window by eight different orientations. Next, the resulting patches are projected onto the Radon space. It is worth noting that prominent valleys in Radon space are created by the vein patches. The curvature values of the veins are used to enhance the vein patterns, which after binarization are extracted in good quality.

Yang et al. [87] proposed an adaptive vector field estimation algorithm for feature extraction from finger vein images. Using Gaussian Weighted Spatial Curve Filtering (GWSCF), they extracted the features from the finger vein images. The same year, Janney et al. [88] in their method used Discrete Wavelet Transform (DWT). Discrete Wavelet Transform decomposes the image into two bands: low-pass components and high-pass components. They state that it is a lossless compression method and does not degrade the quality of an image.

In 2018, Subramaniam and Radhakrishnan [89] developed a biometric authentication system that uses finger, palm, and dorsal vein images. After preprocessing the image, the feature extraction was performed by applying three different transformations: Hilbert–Hung, Radon, and Dual-Tree Wavelet Transform. The three transformations were applied to the images and fused to form a single feature vector.

Depending on the chosen transformation function, the feature extraction can be sensitive to rotation or scaling factors (such as the Radon transformation) or not (the Haar Wavelet). In most cases, though, image transformation techniques have been proven to perform very well, with very low EERs and high accuracy rates. However, these functions require the setting of some parameter values. Table 4 sums up the studies mentioned in this category, showing the key features of each one and the advantages and disadvantages.

### 4.5. Other Feature Extraction Methods

In 2009, Cong-Li et al. [90] used morphological operations to extract the features from a finger vein image. First, a set of boundary points of the image is created. Then, for each boundary point, the image is scanned in various directions by applying the multi-scale top hat transformation to extract the valleys from the image. Eight connected objects are found and labeled on the resulting image. A second segmentation is performed for a specific threshold and the result is optimized by thinning and deburring.

In 2010, Liukui and Zheng [91] extracted the features from a finger vein image by using a tri-value template. Using predefined threshold values, the finger vein image is segmented into three areas: the subject area, the background area, and the fuzzy area. The object area and fuzzy area are then used for matching.

The same year, Mahri et al. [92] used the properties of the Band-Limited Phase Only Correlation (BLPOC) function for finger vein image matching. After preprocessing the image, four sets of horizontally displaced images are created to overcome the finger vein displacement errors. Lastly, the BLPOC function is calculated between the input (displaced and non-displaced) images and the registered images. Xianming et al. [93] proposed a method based on gray valley-shaped region searching, using profile curve valley-shaped characteristics of images to achieve vein feature extraction. By analyzing the characteristics of all the directions of the gray curve, the gray pixels belonging to the valley-shaped region are determined and then all the results for the different directions are overlaid.

Tang et al. [94] introduced an Occurrence Probability Matrix (OPM), which consists of probability values that describe the reliability of each unit in a template. A training set was used to calculate the OPM, which was also used to create a fused template that represents the finger.

Xi et al. [95] applied a combination of Pyramid Histogram of Texture (PHT), Pyramid Histogram of Gray (PHG), and Pyramid Histogram of Oriented Gradients (PHOG) to extract features. The new method, called Pyramid Histograms of Gray, Texture, and Orientation Gradients (PHGTOG), can represent the global spatial layout and local gray, texture, and shape details. The Least Absolute Shrinkage and Selection Operator (LASSO) algorithm is used on sparse weight vectors to train subjects; thus, the selected features are called PFS-PHGTOG. Cao et al. [96], after preprocessing a finger vein image and skeletonizing it, detected minutiae points in the image. A curve tracing algorithm was then deployed and a Modified Include Angle Chain (MIAC) was applied to encode the curves. The feature representing the finger vein junction is a vector that consists of the junction coordinates and the MIAC codes connected with the junction. All the features of the junctions represent a single finger vein.

In 2014, Rajan and Indu [97] used a Fast Retina Keypoint (FREAK) descriptor to extract features from finger vein images. The keypoints are found by first applying a Frangi filter to the finger vein image, and the Features from Accelerated Segment Test (FAST) algorithm is then used to find the keypoints. Liu et al. [98] used the Simple Linear Iterative Clustering (SLIC) method to generate a superpixel and called this procedure superpixel over-segmentation. Superpixel-based features (SPFs) extract the superpixel histogram features and the superpixel distribution features of each finger vein image using statistical techniques.

In 2015, Soundarya et al. [99] combined the Lacunarity and Mandelbrot fractal models to extract the features of the finger vein. The Lacunarity model is based on the Blanker technique and helps to differentiate images that are visually different but have similar fractal dimensions. Later the same year, Jadhav and Nerkar [100] used the Canny edge detector to extract the edges and curves from finger vein images. You et al. [101], after preprocessing, thresholding, and skeletonizing a finger vein image, calculated the Potential Energy Eigenvectors (PEEs) and used features.

In 2017, Bai et al. [102] developed a feature extraction method using a SVM classifier. After the extraction of the features, the finger vein image is classified into background pixels and binary vein patterns. Then, the vein pattern is matched with the vein patterns in databases such as FUSM, ORL, and VP.

In 2018, Banerjee et al. [103] presented a system called ARTeM that uses template matching. The images are preprocessed (ROI extraction, intensity normalization, fuzzy contrast enhancement, CLAHE histogram equalization, and directional dilation) and transformed using an affine transformation model.

A year later, Kovač and Marák [104] proposed an automated identification system that combines fingerprint and finger vein images in the authentication. For the finger veins, the feature extraction is done using both the SIFT and SURF algorithms by performing the first out of five different phases, while the second one is used to detect scale- and rotation-invariant points. Similarly, Meng et al. [105] designed a framework for the calculation of a matching score between two finger vein images by fusing three kinds of features. The features are calculated from pixels matched via a dense SIFT descriptor [106], where a pixel-to-pixel score, an object value optimization function, and texture displacements matrices were calculated. The fusion was performed by using the fusion weights that were learned by a SVM model.

In this category, a number of different methodologies have been proposed over the years for extracting the unique patterns of humans’ finger veins. Generally, it seems that the usage of keypoint detection and descriptor methods have been chosen in most cases. Studies have applied quite a few of the available detectors (SIFT, SURF, FREAK, and FAST), with the classification using both a matching score and a ML model. In the first case, a large dataset is not required to match any given example, but it can become harder to identify in cases of many classes, while the latter can tackle this problem but requires a large dataset for the training process. Table 5 sums up the studies mentioned in this category, showing the key features of each one and the advantages and disadvantages.

## 5. Feature Extraction vs Feature Learning

The feature extraction methods presented in the previous section share the same property of being inspired by the prior knowledge of some application experts. The designers of such feature extraction algorithms need to have knowledge of finger vein anatomy as well as information coding/representation and computer vision skills. This requirement of prior knowledge makes the design of these methods a difficult and demanding task.

One of the reasons for the rise of deep learning is the automation and optimization of the feature extraction procedure. For example, Convolutional Neural Networks (CNNs), which are the most popular deep learning models for computer vision applications, consist of several feature extraction layers before the final decision layers. The feature extraction layers learn to extract optimized feature representations (convolutional kernels) from the training images. In this context, the process of using prior knowledge to extract the useful features from an image has been transformed into a *feature learning* task based on a massive number of training images.

The first attempt to deploy deep learning models in finger vein biometrics was by Radzi et al. [107] in 2016. In this work, a preprocessing procedure was first applied to the finger vein image in order to extract a ROI of 55 × 67 pixels in size. Then, the image was fed to a customized four-layer CNN with a 5–13–50–50 architecture. The performance of this method for an in-house finger vein dataset was very promising. The same methodology was also adopted by Itqan et al. [108] to develop a user authentication application in MATLAB IDE.

In 2017, Hong et al. [109] proposed the application of the pre-trained VGGNet-16 [110] CNN model, which consists of 13 convolutional layers, 5 pooling layers, and 3 fully connected layers (FCLs). Initially, an ROI of 224 × 224 pixels in size that includes the finger vein is detected using the method described in [111] and the difference between the input image and the enrolled image is fed to the CNN for recognition.

The following year, the number of attempts to apply deep learning models increased significantly. More precisely, Yang et al. [112] used stacked the Extreme Learning Machine (ELM) deep learning model and Canonical Correlation Analysis (CCA) [113] to build a multi-modal biometric system, called the S-E-C model, based on face and finger vein biometrics. Firstly, a stacked ELM is used to produce a hidden-layer representation of the finger vein images (along with the face images). Then, the CCA method is used to convert the representation produced by the stacked ELM to a feature vector, which is finally passed through to an ELM classifier.

Kim et al. [114] proposed a multi-modal biometric methodology utilizing the finger shape and finger vein patterns for authentication purposes. The introduced method includes a preprocessing stage for compensating for the in-plane rotation and extracting the ROI of the finger vein. Moreover, this method makes use of an ensemble model consisting of two CNNs, based on the ResNet-50 and ResNet-101 architectures, without the output layer. Hu et al. [115] proposed a customized CNN architecture, called FV-Net, that uses the first seven layers of the pre-trained VGGFace-Net [116] model and the addition of three more convolutional layers that learn the specific vein-like features.

Fairuz et al. [117] proposed a CNN architecture of five convolutional and four fully connected layers, while the input images should be 227 × 227 × 3 pixels in size. They evaluated their model using an in-house dataset of 1560 images. In the same year, Das et al. [118] also used a customized CNN consisting of five convolutional layers, three max-pooling layers, one ReLU, and a Softmax layer. The reported advantage of this model is the ability of the CNN to handle non-square images since the input image has a size of 65 × 153 × 1 pixels and the used kernels are of an optimized size.

In 2019, Xie and Kumar [119] used a Siamese CNN model after image preprocessing, enhancement, and supervised discrete hashing [120] for finger vein identification. They compared the results of different configurations of the Light CNN (LCNN) and the VGG-16 models. On the other hand, Lu et al. [121] presented the CNN competitive order (CNN-CO) local descriptor, which is generated by using a CNN that is pre-trained on ImageNet. After selecting the effective CNN filters from the first layer of the network, the CNN-CO computes the CNN-filtered images, builds the competitive order image, and, lastly, generates the CNN-CO pyramid histogram. Song et al. [122] proposed a modified version of the DenseNet-161 [123] model, which is applied after image preprocessing, restoring of the empty regions, and constructing composite and difference images using the enrolled and input images. Finally, Li and Fang [124] proposed an end-to-end Graph Neural Network (GNN), called FVGNN, consisting of the EmbedNet CNN for feature extraction and the EdgeNet. The authors examined three different types of CNNs for the case of the EmbedNet: VGG-based, ResNet-based, and Inception-based networks.

The next year, Kuzu and Maiorana [125] introduced an ad hoc acquisition architecture comprised of CNNs and RNNs. A CNN was used to extract features from images of four finger veins, which were then fed to a Long-Short Term Memory (LSTM) model, as a sequence, for classification. Noh et al. [126] used both texture images and finger vein shape images to train two CNN models. After stacking the enrolled and input images onto a three-channel image, they fed them into the corresponding CNNs. Each CNN outputs a matching score between the images, which is then corrected with a shift matching algorithm. Lastly, the two scores are fused together to provide the final decision. Cherrat et al. [127], in their finger vein system, used a CNN as a feature extractor combined with a Random Forest model for the classification, while Zhao et al. [128] used a lightweight CNN for the classification and focused on the loss function by using the center loss function and dynamic regularization. Hao et al. [129] proposed a multi-tasking neural network that performs both ROI and feature extraction sequentially, through two branches. The model is similar to the Faster RCNN and makes use of the SmoothL1 loss function for the ROI detection branch and the ArcFace loss functions for the feature extraction branch. Lastly, Kuzu et al. [130] investigated the application of transfer learning by using pre-trained CNN models trained on the ImageNet dataset, with satisfactory results.

It is worth mentioning that the incorporation of deep learning models into finger vein recognition systems is mainly focused on the substitution of the manual feature extraction with an automatic feature learning approach. However, the main disadvantage of these approaches is the need for large datasets, which at this moment are not available, a weakness that is managed by incorporating data augmentation techniques. Studies have made use of transfer learning methodologies without achieving results as good as those from some of the methodologies mentioned in the previous sections. The reason for this is the nature of the images captured from the device, as these types of images have unique characteristics compared with the images included in the ImageNet dataset (which most pre-trained models have been trained on). Despite that, CNNs have been shown to achieve very good results if a large amount of data exists to train them on. Table 6 summarizes the studies mentioned in this category.

## 6. Implementation Aspects

The three basic building blocks for implementing and evaluating a finger-vein-based authentication system are: (1) a set of a sufficient number of fingerprint images (benchmark images), (2) a software framework/library in which the authentication methodology will be developed, and (3) the hardware used by the methodology. These three implementation aspects are considered as areas of decision for each researcher working in the field and for this reason the possible options that exist will be discussed in this section, as they have emerged from the previously presented literature.

### 6.1. Benchmark Datasets

For the development and evaluation of any method of authentication with finger veins, but also of any computer vision application, the use of sets of images commonly used in the literature is required. In a significant number of works discussed in previous sections, in-house image datasets that are not available to the scientific community were used. However, in several papers benchmark datasets were used, the characteristics of which are shown in Table 7.

It is worth noting the software [138] proposed by The Hong Kong Polytechnic University for the synthetic generation of finger vein images.

From Table 7, it can be deduced that the size of the benchmark datasets is small to medium and, although these data seem to be sufficient to train shallow machine learning models, for the case of deep learning models these datasets are not sufficient to provide high accuracy rates. Table 7 brings to light the problem of the non-availability of large datasets able to train deep architectures, e.g., CNNs, and highlights the need to design larger and better-quality benchmark datasets.

### 6.2. Software Frameworks/Libraries

Recently, there has been an increasing trend to develop open-source software libraries to promote the development of a scientific discipline. At the same time, many researchers provide the source codes they use to implement their methodologies through open-source platforms, e.g., GitHub.

In this context, we identified some software implementations of feature extraction methods as well as full finger-vein-based authentication methodologies, which may serve as a good starting point for new researchers in the field and are summarized in Table 8.

### 6.3. Hardware Topologies/Configuration

The last important implementation aspect that has to be considered in developing finger vein authentication systems is the applied hardware topology along with the characteristics of the used additional hardware components. Table 9 summarizes the main hardware topologies proposed in the examined studies, as well as information about the used cameras, camera filters, and NIR LEDs.

Most approaches suggest a (top-down) hardware topology with two different component configurations. According to this topology, a camera and a NIR LED are placed opposite and the finger is placed between them. However, a topology with multiple NIR LEDs and cameras has also been proposed [125]. Table 8 also reveals the high diversity of the NIR LED wavelengths that are deployed towards acquiring more descriptive finger vein images.

## 7. Conclusions and Discussion

This work presents a comprehensive review of the feature extraction methods proposed for finger vein biometrics. This review can be used as a guide for those who are interested and want a clear view of this research field. As this field is currently in the spotlight and there is not as much information as on other biometrics, for example, fingerprints, that can guarantee a low error rate, it could be used as a starting point for newcomers who want to make a breakthrough in the field. Moreover, despite the fact that finger veins have a lower accuracy than other biometric traits, they are worth investigating because they have a number of advantages, such as being very difficult to forge, and a human has more than one finger, which can be used for authentication purposes.

In the past several years, authentication based on finger vein images has seen an improvement as far as the performance goes. The best performance can be seen in the methodologies of feature learning, where deep learning is employed. Those have the best performance on average, with many methodologies achieving over 99% of accuracy, despite the small (for deep learning) datasets available.

Regarding the experiments in the literature, we conclude that most of the studies, especially in the early years, did not evaluate the proposed methodology on publicly available datasets. This is mainly attributed to the fact that some the currently available datasets only became available later on. Moreover, the chosen metric for the performance evaluation varies across studies, with most of the studies presenting the EER, RR, or ROC scores.

As future work, for comparative reasons, it is highly recommended that researchers present their proposed methodology’s performance using the same and more interpretable metrics. Additionally, the splitting of the training and testing set sizes, for those methodologies that apply any type of learning procedure, has to be the same too. In this context, the design of large-scale datasets (big data) that will permit the training and validation of customized CNN models from scratch is of paramount importance towards the development of more reliable finger vein biometric systems.

## Figures and Tables

**Table 1:** Characteristics of the vein-pattern-based feature extraction methodologies.

Ref.	Key Features	Advantages	Disadvantages
[10]	Application of line tracking	Robust against dark images, fast with a low EER (0.14%)	Mismatch increases when veins become unclear
[11]	Application of local maximum curvatures	Not affected by fluctuations in width and brightness, low EER (0.0009%)	Evaluated only with one dataset of 638 images
[12]	Combination of gradient normalization, principal curvature, and binarization	Not affected by vein thickness or brightness, EER of 0.36%	High EER
[13]	Extraction of minutiae with bifurcation and ending points	Used as a geometric representation of a vein, low EER (0.76%)	Tested on a small dataset
[14]	Extraction of local moments, topological structure, and statistics	A Dempster–Shafer fusion scheme is applied	Low accuracy (98.50%)
[15]	Application of Gabor filter banks	Takes into account local and global features, performs well in person identification	Low accuracy (98.86%)
[16]	Application of maximum curvature	Overcomes low contrast and intensity inhomogeneity	High EER (8.93%)
[17]	Extraction of phase and direction texture features	Does not require preprocessing, has a low storage requirement	Robustness in the presence of noise is not studied
[18]	Application of the mean curvature method	Extracts patterns from images with unclear veins, fast with a low EER (0.25%)	Small dataset
[19]	Application of multi-scale oriented Gabor filters	Takes into account local and global features	Low RR (97.60%)
[20]	Application of guided Gabor filters	Does not require segmentation, good against low contrast, illumination, and noise	High EER (2.24%)
[21]	Cryptographic key generation from a contour-tracing algorithm	Small probability of error when the image is altered and robust against minor changes in direction or position	No recognition results presented
[22]	Maximum curvature method, Gabor filter, minutiae extraction	Elimination of false minutiae points	Performance analysis is not reported
[23]	Combination of SURF with Lacunarity	Shows real-time performance	Experimental information is missing
[25]	Application of SVDMM	Performs better than similar works	High EER (2.45%)
[26]	Combination of minutiae extraction and false pair removal	Eliminates false minutiae matching	Low accuracy (91.67%)
[27]	Application of repeated line tracking	Simplicity	Part of a multi-modal system, no results presented
[28]	Combination of multi-scale matched filtering and line tracking	Extracts local and global features	High EER (4.47%)
[29]	Combination of minutiae extraction and curve analysis	Low EER (0.50%)	Low accuracy (92.00%)
[30]	Application of modified repeated line tracking	More robust and efficient than the original line tracking method, fast	Depends heavily on the segmentation result
[31]	Application of gradient boost	Fast, is not affected by roughness or dryness of skin	No results presented
[32]	Curvature through image intensity	Robust against irregular shading and deformation of vein patterns, fast with a low EER	Requires capturing of finger outlines
[33]	Overlaying of segmented vein images for feature generation	Generation of optimal quality templates	Low accuracy (97.14%), small dataset
[34]	Application of neighborhood elimination to minutiae point extraction	Takes into account intersection points, reduced feature vector size	No RR or EER results provided
[35]	Application of Gabor filters	Captures both local orientation and frequency information	No results presented
[36]	Application of different feature extraction methods (maximum and principal curvature, Gabor filters, and SIFT)	Low EER (0.08%)	Fusion of different perspectives needs improvement
[37]	Application of orientation map-guided curvature and anatomy structure analysis	Easy vein pattern extraction, fast, overcomes noise and breakpoints, Low EER (0.78%) and high RR (99.00%)	The width of the vein pattern is not used
[38]	Application of an elliptical direction map for vein code generation	High accuracy (99.04%)	Results depend on parameters
[39]	Combination of KMeans Segmentation with canny edge detection	Low EER (0.015%)	Small dataset
[40]	Application of SLA	Ensemble learning is applied	Low accuracy (87.00%)
[41]	Application of C^2^ code	Takes into account orientation and magnitude information, low EER (0.40%)	Dataset information is missing
[42]	Application of PWBDC	Low storage requirement and effective with a low EER	Low accuracy (98.9%), High EER (2.20%)
[43]	Application of principal curvature using a Hessian matrix	Suitable for FPGA	No results presented
[44]	Application of Spectral Clustering	Takes into account useful vein patterns, a low EER (0.037%)	Selection of an appropriate seed parameter value

**Table 2:** Characteristics of the dimensionality-reduction-based feature extraction methodologies.

Ref.	Key Features	Advantages	Disadvantages
[45]	Application of pattern map images with PCA	Fast and a high identification rate (100%)	High number of feature vectors (40 features), results depend on parameters
[46]	Application of manifold learning	Robust against pose variation, a low EER (0.80%)	Low RR (97.80%)
[47]	Combination of B2DPCA with eigenvalue normalization	Improves upon the original 2DPCA method and other methods	Low RR (97.73%)
[48]	Combination of Radon transformation and PCA	Low FAR (0.008) and FRR (0)	An in-house dataset is used instead of a benchmark one
[49]	Application of linear discriminant analysis with PCA	Very fast and retains the main feature vector	Low Accuracy (98.00%)
[50]	Application of (2D)^2^PCA	High RR (99.17%)	Sample increment with SMOTE
[51]	Comparison of multiple PCA algorithms	Can reach an accuracy of up to 100%	Requires a large training set
[52]	Application of KPCA	High accuracy (up to 100%)	Accuracy depends on the kernel, feature output, and training size
[53]	Combination of KMMC and 2DPCA	Improves upon the recognition time of just KMMC	Very slow recognition time
[54]	Combination of MFRAT and GridPCA	Fast and robust against vein structures, variations in illumination and rotation	Low RR (95.67%)
[55]	Application of pseudo-elliptical sampling model with PCA	Retains the spatial distribution of vein patterns, reduces redundant information and differences	High EER (1.59%) and low RR (97.61%)
[56]	Application of Discriminative Binary Codes	Fast extraction and matching with a low EER (0.0144%)	Requires the construction of a relation graph
[57]	Combination of Gabor filters and LDA	Low EER (0.12%)	Part of a multi-modal system
[58]	Application of multi-scale uniform LMP with block (2D)^2^PCA	Preserves local features with a high RR (99.32%)	Does not retain global features and the EER varies per dataset (high to low)

**Table 3:** Characteristics of the local binary pattern-based feature extraction methodologies.

Ref.	Key features	Advantages	Disadvantages
[59]	Usage of NN for local feature extraction	Very fast and robust against obscure images	High EER (0.13%)
[60]	Alignment using extracted minutiae points	Fast with a low EER (0.081%)	An in-house dataset is used instead of a benchmark one
[61]	Extraction of holistic codes through weighted LBP	Reduced processing time and a low EER (0.049%)	Requires setting of weights
[62]	Combination of LBP and Wavelet transformation	Low EER (0.011%), fast, and robust against irregular shading and saturation	Tested on a small dataset
[63]	Combination of a modified Gaussian high-pass filter with LBP and LDP	Improvement compared with using vein pattern features, a faster processing time, an EER of 0.89%	Not reported
[64]	LBP image fusion based on multiple instances	Simple with low computational complexity and improves the RR on low-quality images	High EER (1.42%)
[65]	Application of PBBM	Removes noisy bits, personalized features, and highly robust and reliable with a low EER (0.47%)	A small in-house dataset is used instead of a benchmark one
[66]	Application of GLLBP	Performs better than other conventional methods on the collected dataset, an EER of 0.58%	Not reported
[67]	Application of MOW-SLGS	Takes into account location and direction information	Low RR (96.00%)
[68]	Application of enhanced BGC (LHBGC)	Fast, a low EER (0.0038%) when using multiple fingers, and robust against noises	Low EER in cases with multiple fingers
[69]	Application of LEBP	Low FPR (0.0129%) and TPR (0.90%)	Low accuracy (97.45%)
[70]	Application of DSLGS	More stable features with better performance than the original	High EER (3.28%)
[71]	Application of CSBC	High accuracy (99.84%) and a low EER (0.16%)	Multi-modal application
[72]	Application of PDVs and AMBP	Solves out-of-sample problems, robust against local changes, and fast with a low EER (0.29%) and a high RR (100%)	Accuracy depends on parameters
[73]	Application of multi-directional PDVs	Outperforms state-of-the-art algorithms with a low EER (0.30%)	Complexity analysis is not reported
[74]	Fusion of vein images with an ECG signal through DCA	Better than two individual unimodal systems, a low EER (0.1443%)	Multi-modal application
[75]	Application of ADLBP	Better describes texture than LBP	Low RR (96.93%), multi-modal application

**Table 4:** Characteristics of the image-transformation-based feature extraction methodologies.

Ref.	Key Features	Advantages	Disadvantages
[76]	Multi-scale self-adaptive enhancement transformation	Very fast, a low EER (0.13%)	Timing performance is not reported
[77]	Usage of the Radon transformation for driver identification	High accuracy rate (99.2%) for personal identification	Tested upon a small dataset
[80]	Embedded system using the HAAR classifier	Fast recognition time and low computational complexity	Accuracy analysis is not reported
[81]	Second generation of wavelet transformation	Fast, a low EER (0.07%)	Dataset and experimental information are missing
[82]	Combination of the Radon transformation and common spatial patterns	Fast, a high RR (100%)	Small dataset
[83]	Usage of Discrete Wavelet Packet Transform decomposition at every sub-band	Improves upon Discrete Wavelet Transform and the original DWPT	Low RR (92.33%)
[84]	Variable-scale USSFT coefficients	High reliability against blurred images	Low RR (91.89%)
[85]	Usage of the Haar Wavelet Transformation	High accuracy (99.80%)	Accuracy highly depends on parameters
[86]	Feature enhancement and extraction using the Radon transformation	Improvement in accuracy in contacted and contactless databases	High EER (1.03%)
[87]	Usage of adaptive vector field estimation using spatial curve filters through effective curve length field estimation	Low EER (0.20%), improves recognition performance compared with other methods	Performance analysis is missing
[88]	Usage of Discrete Wavelet Transform	A hardware device is proposed	Small dataset
[89]	Fusion of the Hilbert–Hung, Radon, and Dual-Tree wavelet transformations	Low EER (0.014%) and improves upon other methods	Three vein images from different parts

**Table 5:** Characteristics of the remaining feature extraction methodologies.

Ref.	Key Features	Advantages	Disadvantages
[90]	Combination of morphological peak and valley detection	Precise details, better continuity compared with others, fast, and robust against noise	Low RR
[91]	Application of tri-value template fuzzy matching	Robust against fuzzy edges and tips, does not need correspondence among points, and has a low EER (0.54%)	A set of parameters needs optimization
[92]	Application of BLPOC	Simple preprocessing, fast with a low EER (0.98%)	A set of parameters needs optimization
[93]	Extraction of profile curve valley-shaped features	Fast, easy to implement, and satisfactory results	No classification results provided
[94]	Application of OPM	Enhances the similarity between samples in the same class	High EER (3.10%)
[95]	Application of PHGTOG	Reflects the global spatial layout and local gray, texture, and shape details and fast with a low EER (0.22%)	Personalized weights for each subject, a low RR (98.90%)
[96]	Feature code generation from a modified angle chain	Fast with a low EER (0.0582%)	Small dataset
[97]	Combination of a Frangi filter with the FAST and FREAK descriptors	Reliable structure and point-of-interest extraction	No classification results provided
[98]	Utilization of superpixel features	Extraction of high-level features	Requires setting of weights for the matching process, a high EER (1.47%)
[99]	Application of the Mandelbrot fractal model	Fast, a low EER (0.07%)	Dataset information is missing
[100]	Application of canny edge detection	Fast	Slow recognition time and a low RR
[101]	Application of Potential Energy Eigenvectors for recognition	Fast and higher accuracy compared with minutiae matching, a low EER (0.97%)	Not reported
[102]	Feature extraction using a SVM classifier	Consistent	Low accuracy rate (98.59%)
[103]	Feature contrast enhancement and affine transformation registration	Improved preprocessing, can reach a RR of 100% and an EER of 0%	Results vary highly
[104]	Combination of the SIFT and SURF keypoint descriptors	Robust to finger displacement and rotation	High EER (6.10%) and a low RR (93.9%)
[105]	Takes into account deformation via pixel-based 2D displacements	Low EER (0.40%)	Low timing performance

**Table 6:** Characteristics of the feature-learning-based methodologies.

Ref.	Key Features	Advantages	Disadvantages
[127]	Application of a reduced complexity CNN with convolutional subsampling	Fast with very high accuracy (99.27%), does not require segmentation or noise filtering	More testing is required
[108]	Application of the smaller LeNet-5	Not reported	Small dataset, low accuracy (96.00%)
[109]	Usage of a difference image as input to VGG-16	Robust to environmental changes, a low EER (0.396%)	Performance heavily depends on image quality
[112]	Application of stacked ELMs and CCA	Does not require iterative fine tuning, efficient, and flexible	Slow with low accuracy (95.58%)
[114]	Application of an ensemble model of ResNet50 and ResNet101	Better performance than other CNN-based models, a low EER (0.80%)	Performance depends on correct ROI extraction
[115]	Application of FV-Net	Extracts spatial information, a low EER (0.04%)	Performance varies per dataset
[117]	Application of a customized CNN	Very high accuracy (99.17%)	Performance depends on training/testing set size, more testing is required
[118]	Application of a customized CNN	Evaluated in four popular datasets	Low accuracy (95.00%), illumination and lighting affect performance
[119]	Application of a Siamese network with supervised discrete hashing	Smaller template size	A larger dataset is needed, a high EER (8.00%)
[121]	Application of CNN-CO	Exploits discriminative features, does not require a large-scale dataset, a low EER (0.93%)	Performance varies per dataset
[122]	Stacking of ROI images into a three-channel image as input to a modified DenseNet-161	Robust against noisy images, a low EER (0.44%)	Depends heavily on correct alignment and clear capturing
[124]	Application of FVGNN	Does not require parameter tuning or preprocessing, very high accuracy (99.98%)	More testing is required
[125]	Combination of a V-CNN and LSTM	Ad hoc image acquisition, high accuracy (99.13%)	High complexity
[126]	Stacking of both texture and vein images, application of CNNs to extract matching scores	Robust to noise, a low EER (0.76%)	Model is heavy, long processing time
[127]	Combination of a CNN, Softmax, and RF	High accuracy (99.73%)	Small dataset
[128]	Application of a lightweight CNN with a center loss function and dynamic regularization	Robust against a bad-quality sensor, faster convergence, and a low EER (0.50%)	The customized CNN needs improvement
[129]	Application of a multi-task CNCN for ROI and feature extraction	Efficient, interpretable results	Performance varies per dataset
[130]	Transfer learning on a modified DenseNet161	Low EER (0.006%), does not require building a network from scratch	Performance varies per dataset

**Table 7:** Characteristics of the benchmark datasets used in the examined studies.

Database Νame	Number of Classes	Number of Fingers	Samples per Finger	Total Size	Image Size	Link (accessed on 17 April 2021)
SDUMLA-HMT [131]	106	6	6	3816	320 × 240	http://www.wavelab.at/sources/Prommegger19c/
UTFV [132]	60	6	4	1440	200 × 100	https://pythonhosted.org/bob.db.utfvp/
MMCBNU_6000 [133]	100	6	10	6000	640 × 480	http://wavelab.at/sources/Drozdowski20a/
THU-FVFD [134]	220	1	1	440	720 × 576	https://www.sigs.tsinghua.edu.cn/labs/vipl/thu-fvfdt.html
PLUSVein-FV3 [135]	60	6	5	1800	736 × 192	http://wavelab.at/sources/PLUSVein-FV3/
VERA [136]	110	2	2	440	665 × 250	https://www.idiap.ch/dataset/vera-fingervein
FV-USM [137]	123	8	6	5904	640 × 480	http://drfendi.com/fv_usm_database/

**Table 8:** Some available software frameworks/libraries.

Ref.	Implementation Link (accessed on 17 April 2021)	Programming Language
[139]	https://gitlab.cosy.sbg.ac.at/ckauba/openvein-toolkit	Python
[130]	https://github.com/ridvansalihkuzu/vein-biometrics	Python
[140]	https://pypi.org/project/xbob.fingervein/	Python
[65]	https://github.com/sohamidha/PBBM	MATLAB
[11]	https://github.com/dohnto/Max-Curvature	C++
[10]	https://github.com/dohnto/Repeated-Line-Tracking	C++
[141]	https://github.com/sandeepkapri/Tri-Branch-Vein-Structure-Assisted-Finger-Vein-Recognition	MATLAB

**Table 9:** Common hardware topologies and configurations.

Topology	Camera Type	NIR LED Wavelength (nm)	Additional Hardware
Top-down NIR LED, camera on the opposite side, with the finger in the middle	Common CCD	700–1000	NIR filter on camera lens (in some cases)
Top-down NIR LED, camera on the opposite side, with the finger in the middle	Common CCD or CMOS camera	760–850	Additional LEDs on opposite sides or an angled hot mirror for extra contrast
Top-down NIR LED array, array of cameras on the bottom	CMOS NIR	860	Diffusing glass on NIR LEDs, a 700 nm long pass NIR filter on the camera array
